# Patient mutations in human ATP:cob(I)alamin adenosyltransferase differentially affect its catalytic *versus* chaperone functions

**DOI:** 10.1016/j.jbc.2021.101373

**Published:** 2021-10-29

**Authors:** Harsha Gouda, Romila Mascarenhas, Shubhadra Pillay, Markus Ruetz, Markos Koutmos, Ruma Banerjee

**Affiliations:** 1 Department of Biological Chemistry, University of Michigan, Ann Arbor, Michigan, USA; 2 Department of Chemistry, University of Michigan, Ann Arbor, Michigan, USA; 3 Department of Biophysics, University of Michigan, Ann Arbor, Michigan, USA

**Keywords:** cobalamin, trafficking, crystal structure, adenosyltranferase, EPR spectroscopy, AdoCbl, 5′-deoxyadenosylcobalamin, ATR, adenosyltransferase, DMB, 5,6-dimethylbenzimidazole, EPR, electron paramagnetic resonance, MCM, methylmalonyl-CoA mutase, Ni(II)-NTA, nickel-nitrilotriacetic acid, TCEP, Tris (2-carboxyethyl) phosphine

## Abstract

Human ATP:cob(I)alamin adenosyltransferase (ATR) is a mitochondrial enzyme that catalyzes an adenosyl transfer to cob(I)alamin, synthesizing 5′-deoxyadenosylcobalamin (AdoCbl) or coenzyme B_12_. ATR is also a chaperone that escorts AdoCbl, transferring it to methylmalonyl-CoA mutase, which is important in propionate metabolism. Mutations in ATR lead to methylmalonic aciduria type B, an inborn error of B_12_ metabolism. Our previous studies have furnished insights into how ATR protein dynamics influence redox-linked cobalt coordination chemistry, controlling its catalytic *versus* chaperone functions. In this study, we have characterized three patient mutations at two conserved active site residues in human ATR, R190C/H, and E193K and obtained crystal structures of R190C and E193K variants, which display only subtle structural changes. All three mutations were found to weaken affinities for the cob(II)alamin substrate and the AdoCbl product and increase *K*_M(ATP)_. ^31^P NMR studies show that binding of the triphosphate product, formed during the adenosylation reaction, is also weakened. However, although the *k*_cat_ of this reaction is significantly diminished for the R190C/H mutants, it is comparable with the WT enzyme for the E193K variant, revealing the catalytic importance of Arg-190. Furthermore, although the E193K mutation selectively impairs the chaperone function by promoting product release into solution, its catalytic function might be unaffected at physiological ATP concentrations. In contrast, the R190C/H mutations affect both the catalytic and chaperoning activities of ATR. Because the E193K mutation spares the catalytic activity of ATR, our data suggest that the patients carrying this mutation are more likely to be responsive to cobalamin therapy.

Vitamin B_12_ or cobalamin is a complex organometallic cofactor that is required by two human enzymes, methionine synthase and methylmalonic-CoA mutase (MCM) (1). B_12_ metabolism is, however, supported by an elaborate pathway comprising multiple chaperones that guide its intracellular journey from the cell surface to two target enzymes (2, 3, 4, 5). Studies on patients with inborn errors of cobalamin metabolism have led to the delineation of seven complementation groups *cbl*A*-cbl*F and *cbl*J in addition to the loci encoding cytoplasmic methionine synthase (*cbl*G) and mitochondrial MCM (*mut*) (6). The cytoplasmic branch of the trafficking pathway leads to the synthesis of methylcobalamin, needed by methionine synthase. The mitochondrial pathway leads to the synthesis of 5′-deoxyadenosylcobalamin (AdoCbl) and includes chaperones encoded by the *cbl*A and *cbl*B loci. The *cbl*A and *cbl*B loci encode a GTPase (7) and adenosyltransferase (ATR) (8), respectively, which play distinct roles in cofactor assimilation, loading, and repair of MCM (9, 10, 11, 12).

ATR (also referred to as MMAB for its causal role in methylmalonic aciduria type B) is a bifunctional protein that doubles as an enzyme and an escort in the mitochondrial trafficking pathway (13, 14) (Fig. 1). Its adenosyltransferase activity converts the inactive cob(II)alamin form of the cofactor to AdoCbl and inorganic triphosphate in the presence of a reductant and ATP (15). ATR binds cob(II)alamin in a “base-off” conformation, in which the dimethylbenzimidazole (DMB) tail is held out of coordination distance to the cobalt. The base in the base-on form of cobalamin refers specifically to DMB. Crystallographic snapshots of the homologous ATR from *Mycobacterium tuberculosis* have revealed the importance of protein and cofactor tail dynamics in controlling cofactor coordination and reactivity (16). In the presence of ATP and cob(II)alamin, the N-terminus of ATR becomes ordered, forming a cup shaped structure that forces the DMB tail into a side pocket. In this ternary substrate complex, cob(II)alamin is bound in an unusual four-coordinate state (13, 17). Axial water ligation is sterically prohibited at the upper face by ATP and by hydrophobic side chains on the lower face. Four coordinate cob(II)alamin renders the cofactor more readily reducible by raising the redox potential of the cob(II)alamin/cob(I)alamin couple by an estimated 250 mV relative to the base-on species (18).

**Figure 1:**
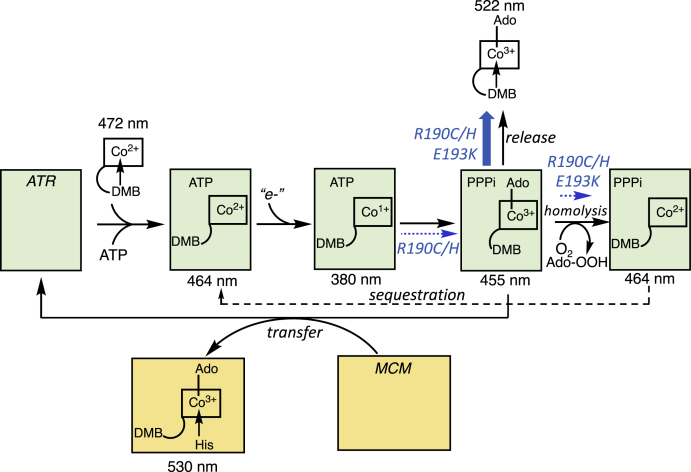
**Multifunctionality of ATR.** ATP binding to ATR (*green*) promotes the binding of cob(II)alamin, which switches from a five-coordinate base-on conformation in solution to four-coordinate base-off conformation in the active site. After the reduction to four-coordinate cob(I)alamin and adenosylation, AdoCbl and PPPi are formed. From here, AdoCbl has three possible fates (*thick blue arrow*). It can be transferred directly to MCM forming six-coordinate base-off/His-on AdoCbl released into solution, or undergo homolysis, potentially leading to its sequestration. The absorption maxima for each of the cobalamin species are indicated. The *blue arrows* denote steps that are either impaired (*dashed*) or accelerated (*thick*) by the R190C/H and E193K mutations in ATR. AdoCbl, 5′-deoxyadenosylcobalamin; ATR, adenosyltransferase; DMB, dimethylbenzimidazole; MCM, methylmalonyl-CoA mutase.

AdoCbl bound to ATR in the ternary product complex has one of the three fates (Fig. 1). It can be transferred directly to MCM (9, 10, 19, 20) where it is bound in a six-coordinate base-off/His on conformation (21). When MCM is not available to accept AdoCbl, ATR catalyzes the homolysis of the newly formed Co-carbon bond in the presence of PPPi, the co-product (10). The homolytic reaction is unusual since ATR catalyzes formation of the Co-carbon bond *via* an S_N_2 displacement reaction at the expense of three high energy phosphate bonds. Because ATR binds cob(II)alamin more tightly than AdoCbl, Co-carbon bond homolysis is speculated to be a strategy for sequestering a high-value cofactor by protecting it from release into the mitochondrial matrix (10). AdoCbl transfer from ATR to MCM is kinetically favored over homolysis. A third fate of AdoCbl is the release into solution, which is accelerated by the R186Q patient mutation (10).

Human MCM is a homodimer, which catalyzes the radical chemistry-based isomerization of methylmalonyl-CoA to succinyl-CoA (22). Occasional inactivation resulting from loss of the deoxyadenosyl intermediate leads to the failure to reform AdoCbl at the end of the catalytic cycle and to accumulation of inactive cob(II)alamin in MCM, which needs repair (23). Cob(II)alamin is translocated from MCM to ATR in a cofactor off-loading process that is driven by GTP hydrolysis catalyzed by the CblA chaperone (9).

Human ATR is a homotrimer with active sites that are located at the subunit interfaces (10, 24). In this study, we have examined the biochemical penalties incurred by patient mutations, R190H/C and E193K (8, 25). ATR mutations are associated with either severe, early onset disease characterized by ketoacidosis, failure to thrive, and encephalopathy or late onset disease with milder presentation (25). Treatment options are limited and include dietary restriction and supplements (*e.g.*, hydroxocobalamin), which has motivated the development of pharmacological chaperones as a therapeutic option (26, 27, 28).

Arg-190 and Glu-193 represent conserved residues located in the active site of ATR (29). Arg-190 makes multiple electrostatic contacts with the ribose oxygen and the α-phosphate in ATP whereas Glu-193 makes a single contact with the C6 adenine amine in ATP and AdoCbl. Our combined kinetic, spectroscopic, and structural studies predict that although the E193K is likely to be catalytically active at physiologically relevant ATP concentrations, the mutation weakens affinity for the product, promoting AdoCbl release into solution *versus* transfer to MCM. In contrast, the R190C/H mutations significantly impair catalytic activity, while also diminishing product affinity, compromising both enzymatic and escort functions of ATR.

## Results

### Kinetic characterization of ATR active site mutants

**Figure 2:**
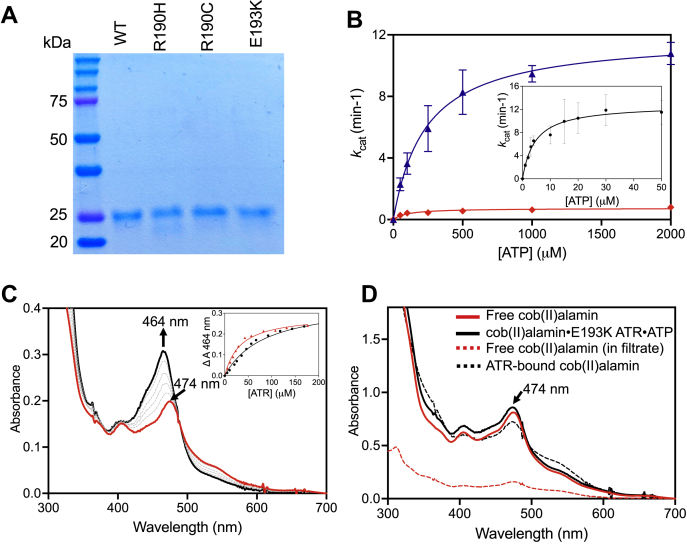
**Characterization of ATR mutants.***A*, SDS-PAGE analysis of WT and mutant ATRs (subunit MW = 23.7 kDa) are used in this study. *B*, dependence of the catalytic activity of WT (*inset*), E193K (*blue*) and R190C (*red*) ATR on the concentration of ATP. The data represent the mean ± SD of 3 independent experiments. *C*, the spectrum of free cob(II)alamin (20 μM, *red*) in buffer A, containing 1 mM ATP and upon addition of increasing concentrations of R190C ATR (*black*, 5–180 μM). The change in the absorption maximum from 474 to 464 nm indicates conversion from five-coordinate to four-coordinate cob(II)alamin. The identical spectral changes were observed with R190C and R190H ATR. *Inset*, dependence of the change in A_464nm_ on the concentration of R190H (*black*) and R190C (*red*) on the concentration of ATR (active site concentration) yielded *K*_D_ values for cob(II)alamin of 38.5 ± 16.5 μM (R190H) and 46.0 ± 10.0 μM (R190C). *D*, the addition of E193K ATR (1.5 mM, *black solid line*) to cob(II)alamin (20 μM, *red solid line*) in buffer A containing 1 mM ATP did not lead to spectral changes. The following filtration using a 10 kDa cut-off filter, 18% of cob(II)alamin was in the filtrate (*red dotted line*) whereas the remainder was protein bound (*black dotted line*). ATR, adenosyltransferase.

The ATR mutants were purified in comparable yield with WT protein and in >95% purity (Fig. 2*A*). Steady-state kinetic characterization revealed that the *k*_cat_ values for E193K and WT ATR were comparable, in contrast to mutations at Arg-190, which resulted in 16-fold (R190C) and 115-fold (R190H) lower activity (Table 1). These data contradict earlier reports that concluded that the E193K and R190H variants are inactive (30, 31). Compared with the WT ATR, the E193K and R190C mutations increased the *K*_M_ for ATP 52-fold and 20-fold, respectively (Fig. 2*B*). The activity of the R190H mutant was too low for the *K*_M(ATP)_ to be reliably determined. Despite repeated attempts, the *K*_D(ATP)_ could not be determined by isothermal titration calorimetry because of the lack of heat change associated with ATP binding to the mutant ATR proteins.

**Table 1:** Steady-state kinetic characterization of ATR active site mutants

ATR	*k*_*cat*_ (min^−1^)	*K*_M_ (ATP), μM	*K*_D(Cob(II)_, μM	*K*_D(AdoCbl)_, μM −PPPi	*K*_D(AdoCbl)_, μM +PPPi
WT	11.5 ± 1.6	4.5 ± 1.1 μM	0.08 ± 0.01	1.0 ± 0.3	0.2 ± 0.1
R190H	0.1 ± 0.1	ND^a^	38.5 ± 16.5	50 ± 5	13 ± 3
R190C	0.7 ± 0.1	92 ± 31 μM	46.0 ± 10.0	40 ± 6	4 ± 1
E193K	10.8 ± 0.6	235 ± 16 μM	ND^a^	120 ± 30	100 ± 20

a ND: not determined.

Although we were unable to determine the *K*_M_ for cob(II)alamin because of technical limitations of the steady-state assay, the *K*_D_ for this substrate was determined. In the presence of ATP, cob(II)alamin binds to ATR in a four-coordinate base-off conformation (17), which is signaled by a blue shift in the absorption maximum from 474 to 464 nm and an increase in absorption intensity (Fig. 2*C*). Although similar spectroscopic changes were observed with the Arg-190 mutants, the *K*_D_ values were 480- and 575-fold larger than for WT ATR (Table 1). In contrast, spectral changes in cob(II)alamin were not observed with E193K ATR (Fig. 2*D*), indicating either greatly weakened affinity or the presence of bound five-coordinate cob(II)alamin. To distinguish between these possibilities, cob(II)alamin (100 μM) was mixed with E193K ATR (500 μM trimer) and ATP (2 mM), and the unbound ligands were separated by centrifugation using a 10 kDa cut-off filter. Although some cob(II)alamin (18 μM) was present in the filtrate, the remainder was bound to ATR (Fig. 2*D*). These data indicate both weakened affinity and stabilization of 5- instead of four-coordinate cob(II)alamin bound to E193K ATR.

### EPR spectroscopy reveals differences in cob(II)alamin binding to ATR mutants

**Figure 3:**
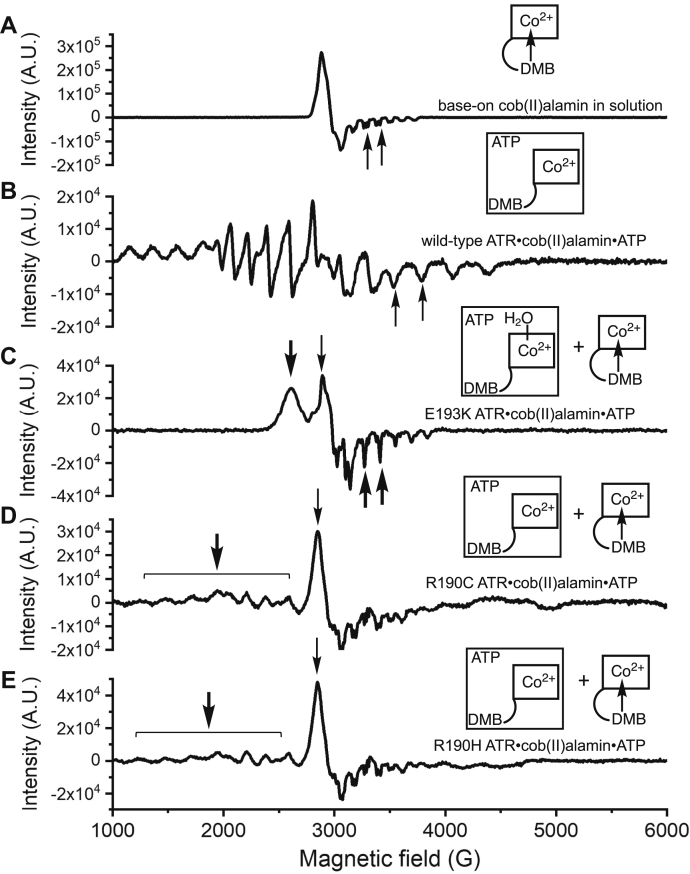
**EPR spectrum of cob(II)alamin bound to ATR in the presence of ATP.***A* and *B*, EPR spectra of cob(II)alamin (300 μM) in buffer A but containing 10% glycerol and ATP (5 mM) in the absence (*A*) or presence (*B*) of WT ATR (200 μM trimer). The shift from an axial to rhombic spectrum and from triplets to singlets in the high field region (*arrows*), signal conversion of cob(II)alamin from five- (free) to four-coordinate (ATR-bound) state. *C*, in the presence of E193K ATR (500 μM trimer) and ATP (5 mM), a mixture of water-coordinated base-off (major, *thick arrows*) and free DMB-coordinated (*thin arrow*) cob(II)alamin was seen. *D* and *E*, in the presence of R190C (*D*) or R190H (*E*) ATR (500 μM trimer) and ATP (5 mM), a mixture of four-coordinate cob(II)alamin (*thick arrow*) and five-coordinate base-on (*thin arrow*) cob(II)alamin were seen. The EPR spectra were recorded at 100 K using the following parameters: 9.27 GHz microwave frequency, 20 mW power, 10 G modulation amplitude, 100 kHz modulation frequency, 5000 G sweep width centered at 3500 G, conversion time 164 ms, and time constant 41 ms. Five scans were collected for each sample. The cartoons depict the cob(II)alamin species found in the samples. ATR, adenosyltransferase; DMB, dimethylbenzimidazole; EPR, electron paramagnetic resonance.

Electron paramagnetic resonance (EPR) spectroscopy allows ready distinction between four- and five-coordinate cob(II)alamin and between an axial nitrogen *versus* oxygen (water) ligand. Interaction between the unpaired electron and the cobalt nucleus (I = 7/2) results in hyperfine splitting into an eight line spectrum whereas nitrogen (I = 1) coordination (*e.g.*, from the DMB base) results in superhyperfine splitting of each of the eight lines into triplets (Fig. 3*A*). The triplet structures are clearly visible in the high field (3250–4000 G) region of the spectrum of free cob(II)alamin. In contrast, the EPR spectrum of cob(II)alamin bound to ATR·ATP is characteristic of four-coordinate cob(II)alamin with singlet hyperfine lines (Fig. 3*B*), as reported previously (17). In the presence of E193K ATR, the EPR spectrum comprises a mixture of five-coordinate base-off (∼80%) and free base-on (20%) cob(II)alamin (Figs. 3*C* and S1). The singlet hyperfine lines in the high-field region of the major component reveal loss of nitrogen coordination. The EPR spectrum confirms that E193K ATR exhibits a weakened affinity for cob(II)alamin, which is bound as a five-coordinate species with a water ligand.

In the presence of R190C/H ATR, a mixture of four-coordinate base-off (75%) and unbound five-coordinate base-on (25%) species was observed (Figs. 3, *D* and *E* and S2). The broad signal resembles that of four-coordinate cob(II)alamin bound to WT ATR (Fig. 3*B*). Together, the EPR spectra indicate that R190C/H ATRs exhibit weakened affinity for cob(II)alamin, which is bound as a four-coordinate species.

### ATR mutations impair AdoCbl binding

**Figure 4:**
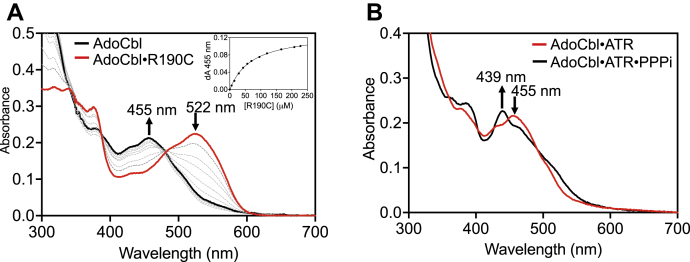
**Binding and weakening of theCo-carbon bond in AdoCbl upon binding to ATR in the presence of PPPi.***A*, free AdoCbl (*red*, 30, μM) in buffer A, undergoes a large spectral shift upon binding to R190C ATR (*black*), signaling conversion from the base-on to the base-off state. Similar changes were seen with WT ATR and the other two mutants. *Inset*. Dependence of the change in absorbance at 455 nm on the concentration of R190C ATR. *B*, PPPi (1 mM) weakens the Co-carbon bond in AdoCbl (30 μM) bound to WT ATR (30 μM trimer) in buffer A as signaled by the shift from 455 to 439 nm. Similar changes were not seen with the mutant ATRs. AdoCbl, 5′-deoxyadenosylcobalamin; ATR, adenosyltransferase.

Free AdoCbl (λ_max_ = 522 nm), which is six-coordinate and base-on, exhibits a large blue shift (λ_max_ = 455 nm) upon binding to ATR, signaling the formation of a five-coordinate base-off species (Fig. 4*A*). Like WT ATR, all three mutants bound AdoCbl in the base-off state but with affinities that were 40- (R190C), 50- (R190H) and 120- (E193K) fold weaker respectively (Table 1). Because the initially formed ATR product complex included PPPi, the *K*_D_ for AdoCbl was also determined in its presence. PPPi (1 mM) decreased the *K*_D_ for AdoCbl 5-fold in WT ATR but had no effect on E193K ATR (Table 1). The R190H and R190C ATRs exhibited 3.9- and 10-fold lower *K*_D(AdoCbl)_ values in the presence of PPPi.

Under anaerobic conditions, PPPi induced weakening of the Co-carbon bond in WT ATR as evidenced by a shift in the absorption maximum from 455 to 439 nm (Fig. 4*B*), as described previously (10). However, a comparable spectral shift was not observed with any of the mutants (not shown), suggesting the loss of PPPi binding, which is required to trigger the spectral change.

### ^31^P NMR spectroscopy reveals weakened affinity of the ATR mutants for PPPi

**Figure 5:**
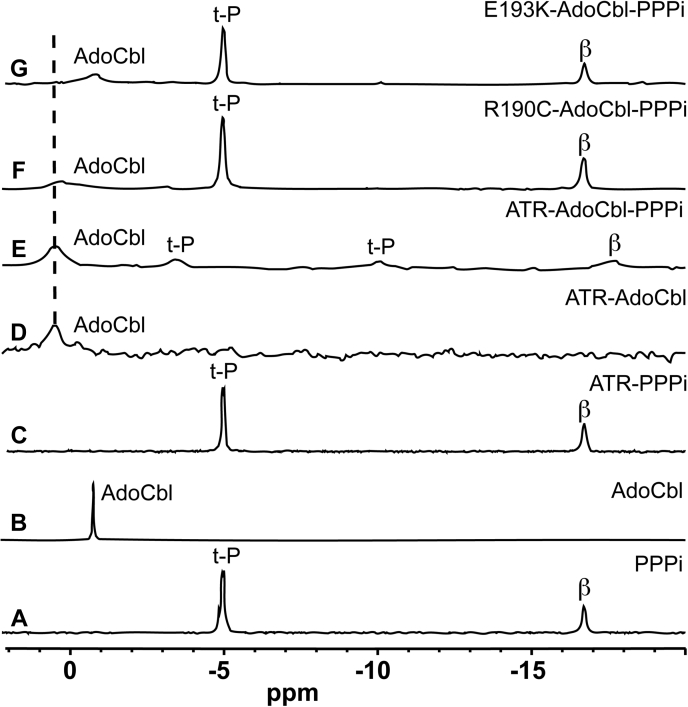
^**31**^**P NMR analysis of PPPi and AdoCbl binding to ATR.***A* and *B*, ^31^P NMR spectra of free 1 mM PPPi (*A*) and 1 mM AdoCbl (*B*) in 50 mM Hepes pH 7.4, 150 mM KCl, 2 mM DTT, and 2 mM MgCl_2_. *C*, the spectrum of PPPi (1 mM) in the presence of WT ATR (1.1 mM) is virtually identical to that of free PPPi, indicating the absence of binding under these conditions. *D*, the spectrum of AdoCbl (1 mM) in the presence of ATR (1.1 mM) shows broadening and a downfield shift of the phosphodiester resonance (Δδ = −1.27 ppm), indicating that the cofactor is bound to ATR. *E*–*G*, the spectra of PPPi (1 mM) and AdoCbl (1 mM) in the presence of WT (*E*), R190C (*F*), or E193K (*G*) ATR. AdoCbl, 5′-deoxyadenosylcobalamin; ATR, adenosyltransferase.

^31^P NMR spectroscopy was used to further investigate the interaction between PPPi and AdoCbl bound to ATR. The proton-decoupled ^31^P NMR spectrum of PPPi (Fig. 5*A*) showed two resonances. The peak at −16.69 ppm (relative to the external 85% H_3_PO_4_ standard) corresponds to the β-phosphorus, which is coupled to the terminal phosphorus nuclei with identical coupling constants. The peak at −4.96 ppm corresponds to the two terminal phosphorus atoms. The resonance for the phosphodiester in the DMB tail in free AdoCbl is observed at −0.74 ppm (Fig. 5*B*). The addition of 1.1 equivalents of WT ATR (active site concentration) to PPPi did not elicit changes in the ^31^P NMR spectrum, indicating that inorganic triphosphate was not bound to the protein under these conditions (Fig. 5*C*). In the presence of ATR, the AdoCbl peak was broadened, consistent with it being bound to the protein and shifted downfield to 0.53 ppm (Fig. 5*D*). The ^31^P nucleus in AdoCbl is too far from the cobalt to be influenced by inductive effects resulting from a base-on to base-off state change (32). An empirical correlation between the ^31^P chemical shift and the smallest O-P-O bond angle in the phosphate ester has been reported (33), which in turn, is sensitive to the ionization state of the acid. Based on this correlation, the downfield shift could indicate an increase in the O-P-O bond angle in AdoCbl bound to ATR.

Several changes in the ^31^P NMR spectrum were observed in a 1.1:1.0:1.0 mixture of WT ATR:AdoCbl:PPPi compared with the spectra for the free ligands (Fig. 5*E*). As described above, the broad peak at 0.53 ppm was assigned to the phosphodiester group in AdoCbl. The β-phosphate (−17.67 ppm) resonance of PPPi was broadened and shifted upfield in the ternary ATR:AdoCbl:PPPi complex. The terminal phosphorus resonances were also broadened but were resolved; one shifted upfield to −10.0 ppm and the other shifted downfield to −3.49 ppm. The chemical shift changes suggest that the electrostatic interactions with active site residues in ATR lead to deshielding of one of the terminal phosphate resonances (Δδ = 1.49 ppm) and shielding of the other (Δδ = −5.0 ppm). The NMR spectrum of WT ATR reveals that AdoCbl increases the affinity for PPPi in the product complex where they are present at a 1:1 stoichiometry.

In the presence of AdoCbl and either R190C or E193K ATR, the PPPi resonances were sharp and identical to those seen for free PPPi (Fig. 5, *F* and *G*). Thus, unlike WT ATR, a 1.1:1.0:1.0 mixture of mutant ATR:AdoCbl:PPPi did not lead to detectable PPPi binding indicating a weakened affinity. The phosphodiester resonance position of AdoCbl showed sensitivity to the active site mutations and was broader and shifted upfield relative to WT ATR. The asymmetric AdoCbl phosphodiester resonance observed in the presence of E193K ATR suggests the possible presence of two conformations.

### Structural basis for ATP binding to R190C ATR

**Figure 6:**
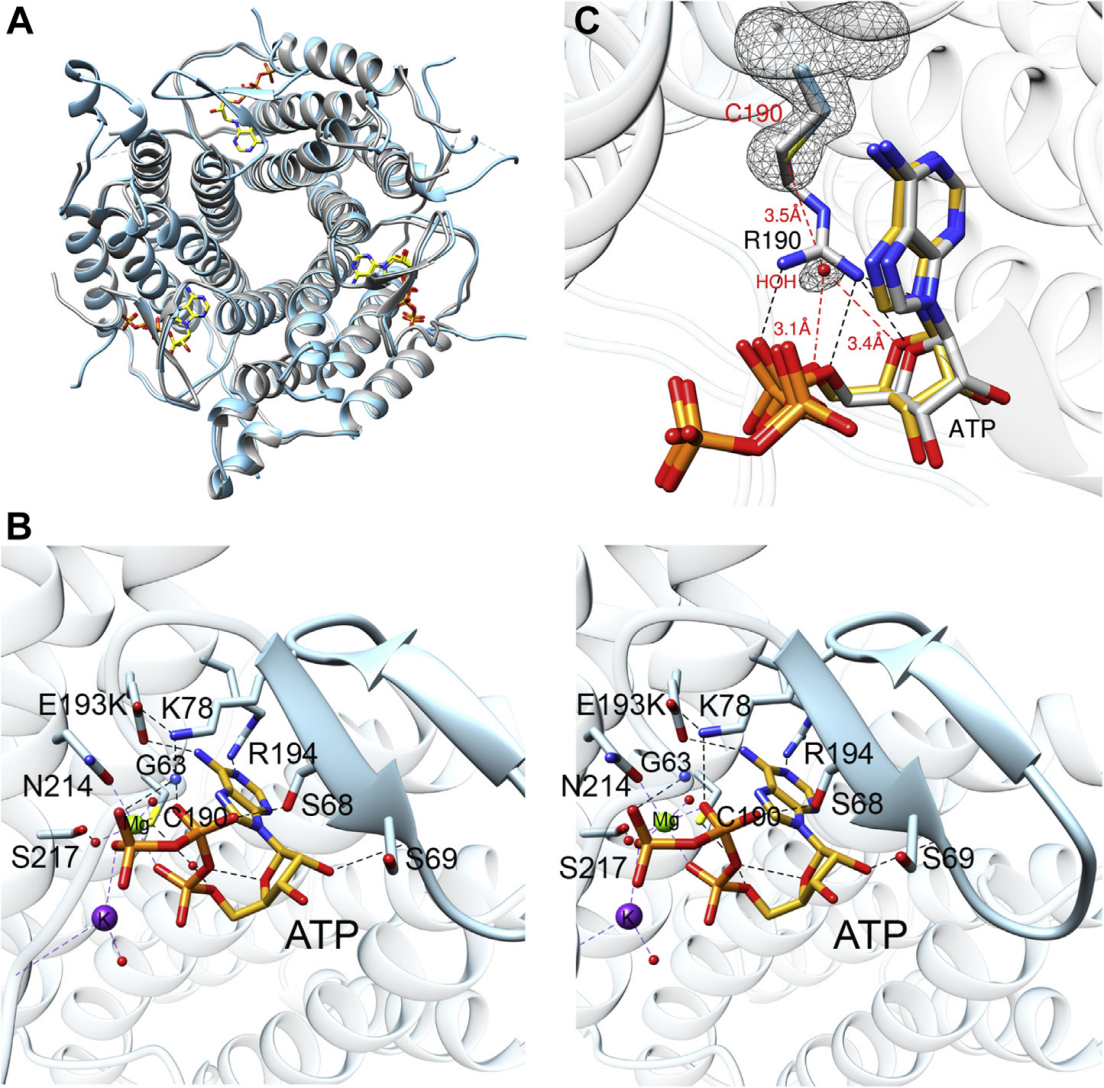
**Structural basis for ATP binding.***A*, an overlay of WT ATR·ATP (PDB code: 2IDX) (*gray*) and R190C ATR·ATP (*blue*). *B*, the stereo view of ATP binding to ATR R190C, which orders residues 68 to 80 into a β-hairpin (*pale blue*). Hydrogen bonds between ATP (*gold sticks*) and side chain residues are shown by dashed lines. *C*, an overlay of the ATP binding site in WT ATR·ATP (*gray*) and R190C ATR·ATP (*gold*). Fo-Fc omit maps (2.5 σ) of Cys-190 and the bridging water are shown as a *gray mesh*. ATR, adenosyltransferase.

The crystal structure of ATP bound to R190C ATR (Table 2) was solved by molecular replacement at 1.5 Å resolution, using the structure of WT human ATR with ATP (PDB code: 2IDX) (24). An alignment of these two structures showed that the R190C mutation did not elicit a major structural change in the protein (C_α_ RMSD = 0.5) (Fig. 6*A*). All six monomers in the asymmetric unit were identical and ATP could be modeled with an occupancy of one per monomer. The structure of R190C ATR·ATP represents the highest resolution structure reported so far for human ATR. In this structure, a complete ordering of loops, except for the first 50 residues, was observed. As seen previously (16), ATP induced ordering of residues 68 to 80 into a β-hairpin, forming the roof of the active site (Fig. 6*B*). An overlay of the ATP binding sites shows that many of the interactions that stabilize ATP binding are retained in the R190C ATR. These include hydrogen bonds between the adenine moiety and the side chains of Glu-193 and Arg-194 and interactions between the PPPi moiety and Lys-78, Ser-217, Asn-214, Gly-63, and Ser-68 either directly or *via* coordination to Mg^2+^ or K^+^.

**Table 2:** Crystallographic data for ATR patient mutations

Data collection and refinement statistics	R190C·ATP	R190C·AdoCbl	E193K·AdoCbl
Beamline	APS, GMCA	APS, LS-CAT	APS, LS-CAT
Wavelength (Å)	1.033	1.033	1.033
Temperature (K)	100	100	100
Space group	P 1 21 1	H3	H3
Cell dimension			
α, β, γ (°)	90, 91.3, 90	90, 90, 120	90, 90, 120
a, b, c (Å)	77.1, 75.7, 95.8	121.8, 121.8, 171.6	121.8, 121.8, 169.7
Resolution (Å)	47.9–1.5 (1.53–1.5)	39.75–1.85 (1.89–1.85)	33–2.1 (2.16–2.10)
*R*_merge_ (%)	6.7 (107)	9.9 (118)	11.8 (78)
*R*_meas_ (%)	7.2 (116)	10.4 (125)	12.7 (94)
*R*_pim_ (%)	2.8 (45)	3.2 (42)	4.7 (52)
<I/σ>	12.8 (1.8)	12.1 (1.6)	8 (1.3)
CC (½)	1.0 (0.66)	0.99(0.69)	0.99 (0.55)
Completeness (%)	99.9 (99.9)	99.8 (99.2)	96 (75.6)
Multiplicity	6.6 (6.6)	10.6 (8.6)	6.2 (3.2)
No. reflections	1169822 (57580)	857285 (39439)	325031 (10656)
No. unique reflections	176322 (8753)	80886 (4590)	52113 (3363)
Overall B (Å^2^) (Wilson plot)	20.4	34.3	40.5
Resolution range	40.49/1.5	36.2–1.85	32.9–2.1
Number of reflections (work/test)	176280/8845	80862/4077	52063/2625
*R*_work_/*R*_free_ (%)	14.9/18.4	17.7/20.2	18.5/21.7
No. of atoms			
Protein	8649	4639	4632
Water	752	300	139
Ligand: B12	NA	364	364
Ado	NA	72	72
ATP	30	NA	NA
B-factors(Å^2^)			
Protein	31.0	41.6	49.1
Ligand: B12	NA	40.3	45.1
Ado	NA	42.6	45.0
ATP	20.8	NA	NA
Water	41.7	46.9	47.2
Rmsd deviations			
Bond lengths (Å)	0.005	0.007	0.007
Bond angles (°)	0.893	0.861	0.889
Ramachandran plot (%)			
Favored, allowed, outliers	98.9, 1.1, 0	98.1, 1.8, 0.2	98.1, 1.9, 0
MolProbity score (percentile)	0.86 (100)	1.3 (98)	1.2 (100)
PDB code	7RUT	7RUU	7RUV

Although the overall mode of ATP binding is unperturbed, the R190C mutation leads to the loss of multidentate electrostatic interactions between Arg-190 and ATP, possibly contributing to the higher *K*_M(ATP)_ value and in turn, the higher *K*_D_ values for cob(II)alamin (in the presence of ATP) and AdoCbl (±PPPi) (Table 1). These data are consistent with a role for ATP in facilitating cobalamin binding by stabilizing the active site architecture. Interestingly, the salt bridge between Arg-190 and the ribose oxygen is replaced by a hydrogen bond between Cys-190 and the ribose oxygen mediated *via* a bridging water (Fig. 6*C*). The salt bridges between Arg-190 and the α-phosphate oxygens are however, lost. The position of the C5′ carbon, which is attacked by cob(I)alamin, is unchanged in the crystal structure of the R190C mutant. Although our attempts to crystallize E193K ATR with ATP were unsuccessful, the structure of WT ATR·ATP allow us to predict its role in ATP binding. Glu-193 forms a salt bridge with the side chain of Lys-78 (Fig. 6*B*) in the β-hairpin roof of the active site. In addition, the side chain of Glu-193 forms a salt bridge with the C6 amino group of the adenine moiety of ATP.

### Structural basis for AdoCbl binding to R190C and E193K ATR

**Figure 7:**
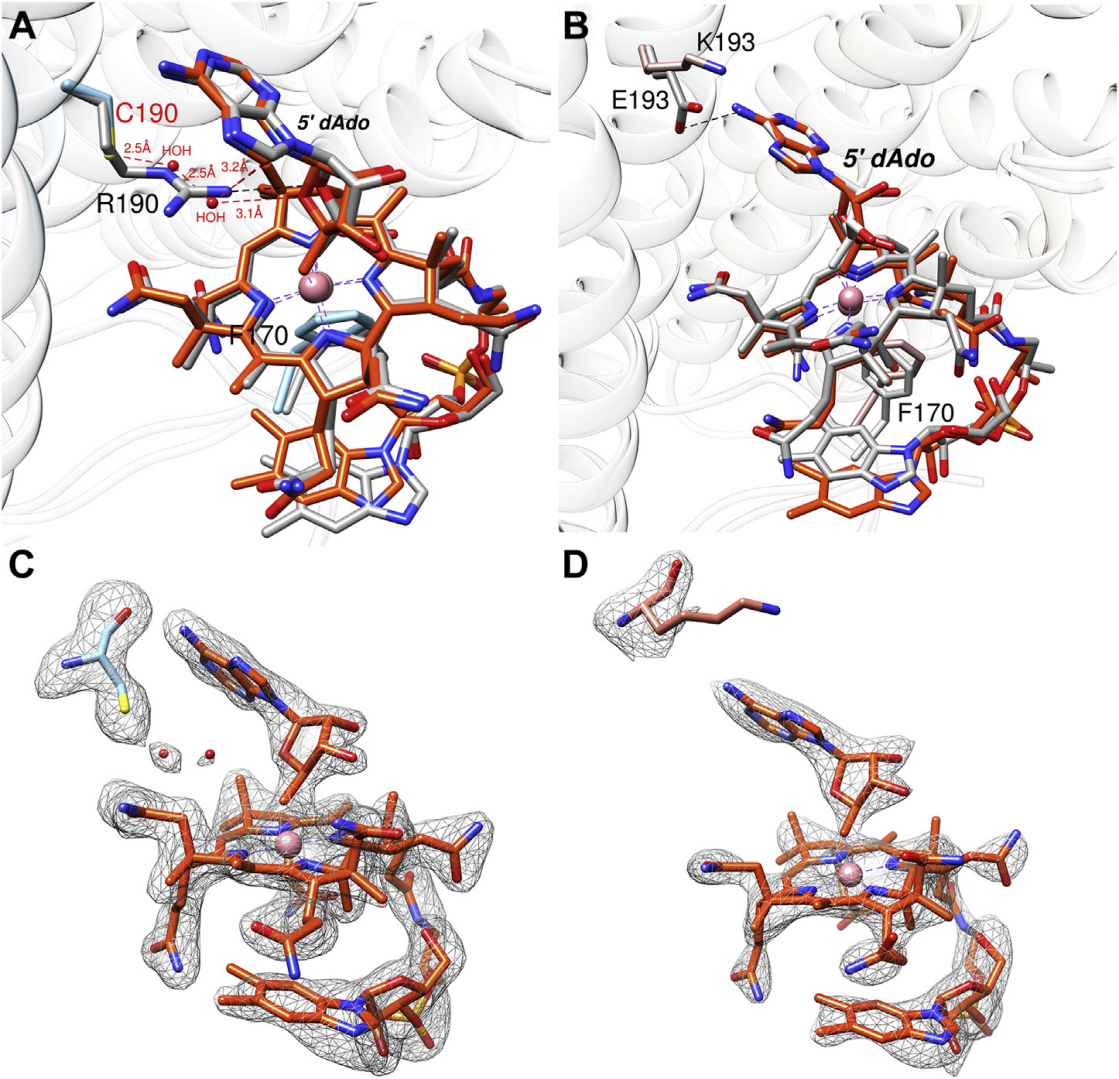
**Structural basis for AdoCbl binding.***A*, the overlay of WT ATR·AdoCbl (*gray*, PDB code: 6D5X) and R190C ATR·AdoCbl (*blue*) shows subtle difference in the position of the ribose ring. The salt bridge between R190 and the Ado moiety (*dashed black lines*) are replaced by hydrogen bonds between C190 and two bridging waters (*dashed red lines*). *B*, an overlay of ATR·AdoCbl (WT *gray*, PDB code: 6D5X) and E193K ATR·AdoCbl (*pink*). *C* and *D*, Fo-Fc omit maps of AdoCbl (*orange*), Cys-190 (*blue*), bridging waters (*red spheres*) (*C*) and Lys-193 (*salmon*, *D*) at 2.5 σ shown in *gray mesh*. AdoCbl, 5′-deoxyadenosylcobalamin; ATR, adenosyltransferase.

To gain structural insights into how the mutations impact AdoCbl binding, R190C and E193K ATR were cocrystallized with AdoCbl and PPPi, and the structures were solved by molecular replacement, as described above. Electron density that could be modeled as AdoCbl but not PPPi was present in the 1.8 Å structure of R190C and 2.2 Å structure of E193K ATR·AdoCbl. Both had four identical chains per asymmetric unit with an occupancy of one AdoCbl per monomer. Structural overlays revealed C_α_ RMSD values of 1.6 Å and 1 Å for WT ATR·ATP *versus* R190C·AdoCbl and E193K·AdoCbl, respectively, indicating that the mutations do not induce major conformational changes in the overall structure. More of the N-terminus was disordered, and both structures were resolved after residue-79, as previously seen with WT ATR·AdoCbl (10). AdoCbl was bound in the base-off conformation with Phe-170 positioned at a distance of 3.6 Å from the cobalt atom (Fig. 7, *A* and *B*). On the upper axial surface, the C5′ carbon of the deoxyadenosyl moiety was 2.1 Å away from the cobalt, which is identical to that observed in WT ATR (7).

An overlay of the WT and R190C ATR structures revealed subtle differences in the position of the ribose ring (Fig. 7*A*). In WT ATR, the side chain of Arg-190 interacts *via* salt bridges to the ribose oxygen and the C6 amino group of the deoxyadenosine moiety. The mutation replaces the salt bridges by a hydrogen-bonding network between Cys-190 and the ribose oxygen that is mediated *via* two water molecules.

An overlay of the WT and E193K ATR structures revealed only subtle differences in the orientation of the deoxyadenosyl moiety (Fig. 7*B*). Indeed, structural changes that might explain the profoundly lower affinity of E193K ATR for AdoCbl (±PPPi) were not obvious in the crystal structure. It is likely that the E193K mutation impacts protein dynamics by destabilizing the β−hairpin roof and, therefore, the active site architecture.

## Discussion

Disease causing mutations represent Nature’s map of residues that are functionally important. In this study, we have focused on three variants at two active site residues in human ATR that have been identified in patients with the MMAB type of methylmalonic aciduria (8). The autosomal recessive nature of this disease and, therefore its rarity, leads to the prevalence of compound heterozygosity, that is, each allele in an affected individual harbors a different mutation. Heterozygosity limits the ability in most cases to make direct correlations between genotype and disease phenotype (*e.g.*, age of onset, severity, and B_12_ responsiveness). Thus, characterization of the biochemical penalties associated with individual mutations could help inform decisions on whether or not patients might be responsive to B_12_ supplementation or other therapeutic strategies, for example, with stabilizing pharmacological chaperones (26, 27). In this study, we demonstrated how pathogenic mutations at two conserved active site residues differentially affect the dual functions of ATR as a catalyst and a chaperone at physiologically relevant concentrations of its substrate.

The chaperone function of ATR in the mitochondrial B_12_ trafficking pathway is supported by studies demonstrating that AdoCbl is directly loaded from ATR to MCM whereas cob(II)alamin is off-loaded in the reverse direction for repair (9, 19, 22). Indeed, to protect against cofactor release in the absence of MCM, ATR harbors the unusual capacity to break the Co-carbon bond in AdoCbl in the newly formed product complex with PPPi (Fig. 1). The resulting cob(II)alamin is more tightly bound than AdoCbl in the presence of the abundant cosubstrate, ATP (10). The cellular relevance of this sacrifical Co-carbon bond homolysis strategy is supported by our analysis of the E193K mutation, which has a greatly reduced affinity for PPPi and fails to trigger homolysis. Despite the 52-fold increase in the *K*_M(ATP)_ value to 235 ± 16 μM, the reaction rate of E193K ATR is unlikely to be affected at cellular ATP concentrations that are in the millimolar range (34). Instead, as with WT ATR, the reaction rate would be determined by cob(II)alamin concentration.

Unlike WT enzyme, the UV-visible spectrum of cob(II)alamin bound to E193K ATR·ATP (Fig. 2*D*) indicates that it is five-coordinate. The EPR data on the other hand revealed that the DMB nitrogen ligand present in free five-coordinate cob(II)alamin is substituted by a water ligand in the E193K ATR·ATP·cob(II)alamin complex (Fig. 3*C*). The E193K mutation dramatically decreases the affinity for AdoCbl and renders it insensitive to the presence of PPPi (Table 1). Although Glu-193 makes a single electrostatic interaction with the amino group of the adenine moiety, it plays a role in stabilizing the β-hairpin roof over the active site *via* its interaction with Lys-78 (Fig. 6*B*). The substitution of a negative by a positive charge at this position is likely to affect loop dynamics.

Although the E193K mutation does not impact enzyme activity under *V*_max_ conditions, it appears to accelerate product release. It is important, however, to note that if ATR-bound cob(II)alamin remains five-coordinate in the presence of its redox partner, it would lower the redox potential relative to the WT enzyme, which binds the cofactor in a four-coordinate state. Although the use of a strong reductant (titanium citrate) in the *in vitro* assay could have masked this effect, kinetic coupling between a difficult reduction and a favorable adenosylation reaction could nevertheless provide the driving force for the reaction. The affinity of PPPi to ATR·AdoCbl is greatly diminished in the 1:1:1 product complex and is in fact, not observed by ^31^P NMR spectroscopy (Fig. 5*G*). These data indicate loss of the cofactor sequestration and concomitant gain of the cofactor release route, leading to its dilution in solution (Fig. 1). Hence, the E193K mutation is predicted to primarily impair the chaperone function of ATR by weakening its affinity for PPPi and AdoCbl in the ternary product complex.

In contrast to Glu-193, Arg-190 makes multiple contacts with ATP and the adenosine moiety of AdoCbl (Figs. 6*C* and 7*A*). Arg-190 is, therefore, important not only for positioning the substrate but also likely to be important for enhancing the leaving group potential of PPPi. In addition, Arg-190 could help stabilize the developing negative charge on the C5′ carbon in the transition state for the nucleophilic attack of cob(I)alamin on ATP. Interestingly, some, but not all of these interactions are compensated for *via* a bridging water molecule as seen in the crystal structure of R190C ATR (Fig. 6, *B* and *C*). Although we were unable to obtain the crystal structure of R190H ATR, the bulky imidazole side chain is likely to preclude a similar compensatory change leading to a more drastic loss of function. The mutations at Arg-190 lead to reduced reaction rate (16-fold (R190C) and 115-fold (R190H)), increased *K*_M_ for ATP, and decreased affinities for cob(II)alamin, AdoCbl, and PPPi (Table 1 and Fig. 5*F*). These pleiotropic effects impair catalytic activity and, like the E193K mutation, are predicted to impair the chaperone activity of ATR by accelerating product release to solution.

The R190C/H mutants have been described as destabilizing as their melting temperatures (∼56.7 °C) are below that of WT ATR (61.4 °C) although, well above the ambient cellular temperature (27). Our characterization of these mutants on the other hand localize the biochemical penalties to weakened substrate and product binding, impacting both catalysis and chaperone activities, as discussed above.

In summary, characterization of the pathogenic mutations at two active site residues predict different bases for the consequent loss of function. The WT like catalytic activity of the E193K variant and predicted impairment of its cofactor delivery activity to MCM lend cellular relevance to this chaperone function. Our biochemical data predict that patients carrying the E193K mutation are more likely to be responsive to B_12_ therapy.

## Experimental procedures

### Materials

AdoCbl, ATP, GTP, PPPi, and dithionitrobenzoic acid were from Sigma-Aldrich. The primers used for mutagenesis were purchased from Integrated DNA Technologies. IPTG and Tris (2-carboxyethyl) phosphine (TCEP) were from gold biotechnology. Nickel-nitrilotriacetic acid (Ni(II)-NTA) resin was from Qiagen.

### Purification of WT and mutant ATR

The expression construct for recombinant human full-length and ΔN18 ATR in a pET-28b vector containing a thrombin-cleavable N-terminal His-tag was transformed into BL21 (DE3) *E. coli* cells*.* A single transformant was inoculated into 100 ml of LB medium containing 50 μg/ml kanamycin and was grown overnight at 37 °C. Approximately, 10 ml of the culture was inoculated into 1 l of LB and, ATR expression ATR was induced with 50 μM IPTG when cells reached an absorbance at 600 nm of 0.6. The cells were grown overnight at 20 °C and harvested the next day by centrifuging at 5500*g* for 20 min.

The cell pellet was resuspended in lysis buffer (50 mM Tris pH 8.0, containing 300 mM NaCl, 10 mM imidazole, and 1 mM TCEP). The cells were disrupted by sonication, and the lysate was centrifuged at 38,500*g* for 20 min at 4 °C. The supernatant was loaded onto a Ni(II)-NTA (5 × 2.5 cm) column, and ATR was eluted using a 200 ml linear gradient (10–200 mM imidazole) in the lysis buffer. The N-terminal His-tag was cleaved with thrombin (5 units/mg protein) during an overnight dialysis at 4 °C against lysis buffer. ATR was loaded onto a second 25 ml Ni(II)-NTA column and eluted using lysis buffer. Thrombin was removed by loading protein on a 5 ml benzamidine column (GE healthcare) and eluted with 25 mM Hepes pH 7.4, 500 mM NaCl, and 1 mM TCEP. ATR was dialyzed overnight against 50 mM Hepes pH 7.5, containing 150 mM KCl, 2 mM MgCl_2_, 2 mM TCEP, and 5% glycerol (buffer A) and stored at −80 °C until use.

The desired patient mutations were generated using the following forward primers and reverse primers with complementary sequences.

R190H-5′-GCCGTGTGVCATCGGGCCGAG-3′

R190C-5′-CTCGGCCCGACAGCACACGGC -3′

E193K: 5′- TGCCGCCGGGCCAAAAGACGTG TGGTG- 3′

The mutations were verified by nucleotide sequence analysis (DNA sequencing core facility, Michigan Medicine). The mutant ATR proteins were purified, as described for WT ATR and were obtained with yields of 25 mg (WT), 20 mg (R190C), 25 mg (R190H), and 20 mg (E193K) per liter of culture.

### AdoCbl binding to ATR

Unless noted otherwise, all titration experiments were performed in buffer A. AdoCbl binding was monitored by adding increasing concentrations of WT or mutant ATR to 30 μM AdoCbl in buffer A at 20 °C. The spectrum was recorded 5 min after each addition. The concentration of bound AdoCbl was determined using Δε_522nm_ = 8.0 cm^−1^ mM^−1^. The change in absorbance at 455 nm (corresponding to base-off AdoCbl bound to ATR) was plotted *versus* ATR concentration and fitted using the Dynafit software (BioKin) (35). Equation 1 (where A is the absorbance at 455 nm following each aliquot of ATR, ΔA_max_ is the maximal change in absorbance at 455 nm upon binding of AdoCbl to ATR, [E]_t_ is the concentration of ATR active sites, and [L]_t_ is the total AdoCbl concentration) was used to determine the dissociation constant for AdoCbl.

(1) $$A=\Delta A_{\text{max}}\times \frac{\left({\left[E\right]}_{t}+{\left[L\right]}_{t}+K_{d}\right)+\sqrt{{\left({\left[E\right]}_{t}+{\left[L\right]}_{t}+K_{d}\right)}^{2}-4{\left[L\right]}_{t}{\left[E\right]}_{t}}}{2{\left[L\right]}_{t}}$$

The effect of PPPi on the *K*_D_ for AdoCbl was determined by performing the above titration in the presence of 1 mM PPPi under anaerobic conditions.

### Cob(II)alamin binding to ATR

Cob(II)alamin binding to ATR was determined by the titrations performed in the anaerobic chamber (<0.2 ppm O_2_ levels). The increasing concentrations of WT or mutant ATR were added to 30 μM cob(II)alamin and 1 mM ATP in buffer A. The concentration of bound cob(II)alamin was determined using Δε_474nm_ = 5.1 cm^−1^ mM^−1^. The change in absorbance at 464 nm, corresponding to the base-off ATR-bound cob(II)alamin was plotted against ATR concentration, and the data were analyzed using Equation 1, as described above.

### AdoCbl synthesis by ATR

AdoCbl synthesis was determined by mixing cob(II)alamin (40 μM) in buffer A at 20 °C with ∼10 equivalents of titanium citrate under strictly anaerobic conditions. The reaction was started by addition of ATP (1 mM) and ATR (1 μM trimer concentration). The concentration of cob(I)alamin formed by reduction of cob(II)alamin was estimated using ε_386nm_ = 28.0 cm^−1^ mM^−1^. The rate of AdoCbl synthesis was determined by measuring the change in absorbance at 522 nm using Δ ε_522nm_ =5.06 mM^−1^ cm^−1.^

### EPR spectroscopy

EPR spectra were recorded on a Bruker EMX 300 equipped with a Bruker 4201 cavity and a Varian liquid nitrogen cryostat. The temperature was controlled by an Oxford Instruments ITC4 temperature controller. The samples were prepared inside an anaerobic chamber (O_2_ < 0.3 ppm) and contained 300 μM cob(II)alamin, 5 mM ATP and WT ATR (200 μM trimer) or 500 μM (trimer) E193K, R190C or R190H ATR in 50 mM Hepes pH 7.5, 150 mM KCl, 2 mM MgCl_2_, 2 mM TCEP, and 10% glycerol. The following instrument settings were employed: microwave frequency, 9.27 GHz; power, 2.0 mW; modulation amplitude, 1.0 mT; modulation frequency, 100 kHz; temperature, 100 K. Five scans were acquired for each sample. A buffer spectrum was subtracted from the sample spectrum. The EPR simulations were performed using the EasySpin program (36).

### ^31^P NMR spectroscopy

The samples were prepared in an anaerobic chamber and contained: PPPi (1 mM), WT, R190C or E193K ATR (1.1 mM active sites), MgCl_2_ (2 mM), AdoCbl (1 mM) in 50 mM Hepes buffer pH 7.5 containing 150 mM KCl and 2 mM DTT at room temperature. The samples were transferred to 5-mm (internal diameter) NMR tubes (Wilmad-Glass), and the final volume was brought up to 0.4 ml with 90% water and 10% D_2_O (Cambridge Isotope Labs).

^31^P-decoupled NMR spectra were collected at 242.5 MHz on a Bruker AVANCE NEO spectrometer equipped with a prodigy cryoprobe locked to D_2_O. All the NMR experiments were carried out at 25 ^°^C using 12 μs 90° pulse widths, 8192 data points, and 4096 scans with 0.8 s recycle time. The chemical shifts were reported on proton-decoupled spectra by Gaussian line fits to the singlet resonances, relative to an external standard of 85% H_3_P0_4_.

### Crystallography and structure refinement

An N-terminal truncated variant of human ATR (ΔN18) was generated using the following forward primer sequence: 5′-CGCGGCAGCCATATGCCCAAGATTTAA CC-3′ along with a reverse primer with the complementary sequence. WT and the R190C variants of ΔN18-ATR and full-length E193K ATR were purified, as described above followed by a final purification using size-exclusion chromatography (Superdex 200, 120 ml, GE Healthcare) in buffer A.

The complexes of E193K and ΔN18-R190C ATR with AdoCbl were prepared by mixing 0.5 mM protein, 1 mM AdoCbl, and 25 mM PPPi in buffer A and used for screening crystallization conditions in the dark. E193K ATR crystals appeared in a drop containing no well solution, whereas the ΔN18-R190C crystals appeared in well solution containing 30% (v/v) 2-methyl-2,4-pentanediol, 100 mM sodium acetate pH 4.6, and 20 mM calcium chloride at 20 °C. The R190C ATR complex with ATP was prepared by adding 5 mM ATP to the protein (15 mg/ml) in buffer A and the crystals appeared in a drop containing 30% (v/v) 2-propanol, 100 mM Tris pH 8.5, and 30% (w/v) PEG 3350. The crystals with the best morphology were obtained by the sitting drop vapor diffusion method from a 1:1 or 2:1 mixture of protein to well solution in 0.9 μl drops. For data collection, the crystals were soaked in a cryoprotectant solution (well solution containing 10–20% v/v glycerol) and flash cooled in liquid nitrogen.

The data sets for E193K ATR with AdoCbl, and R190C ΔN18-ATR with AdoCbl were collected at the LS-CAT (21 ID-D) and for R190C ΔN18-ATR with ATP were collected at GMCA (23 ID-B) at the APS Argonne National Laboratory. Diffraction data were collected at a wavelength of 1.03 Å and 100 °K using a Dectris Eiger 9M detector and Dectris Eiger 16M detector, respectively. The data were indexed and integrated using Xia 2 DIALS (37) or XDS (38) and scaled in Aimless (39). All the structures were solved by molecular replacement using Phaser (40) in the CCP4 program suit. The initial search model was the previously published structure of ATR (PDB code: 2IDX). The crystals of E193K with AdoCbl were of the space group H3 (121.8, 121.8, 169.7, 90, 90, and 120) with four chains per asymmetric unit. The crystals of R190C ΔN18-ATR with AdoCbl were of the space group H3 (121.8, 121.8, 171.6, 90, 90, and 120) with four chains per asymmetric unit. The crystals of R190C ΔN18-ATR with ATP were of the space group P 1 21 2 (77.1, 75.7, 95.8, 90, 91.3, and 90) with six chains per asymmetric unit. Iterative rounds of model building and refinement were performed with COOT and Phenix (41, 42). Ligand restraints were generated in *eLBOW* (43). The geometric quality of the model was assessed in *MolProbity* (44). The models were analyzed, and the structure figures were generated using UCSF Chimera (45).

## Data availability

All data are contained within the article and in the supplemental section. The coordinates for the ATR structures (PDB 7RUT, 7RUU, and 7RUV) have been deposited in the Protein Data Base.

## Supporting information

This article contains supporting information.

## Conflict of interest

The authors declare that they have no conflicts of interest with the contents of this article.
