# Supplementary material for: Patient mutations in human ATP:cob(I)alamin adenosyltransferase differentially affect its catalytic versus chaperone functions
Source: J Biol Chem. 2021 Oct 29;297(6):101373. doi: 10.1016/j.jbc.2021.101373 (PMC8633584; doi:10.1016/j.jbc.2021.101373)
Supplement: Figures S1 and S2 [file mmc1.docx]

**Patient mutations in human ATP:cobalamin adenosyl transferase differentially affect its catalytic versus chaperone functions**

Harsha Gouda^1†^, Romila Mascarenhas^1†^, Shubhadra Pillay^1^, Markus Ruetz^1^, Markos Koutmos^2,3^ and Ruma Banerjee^1^*

^1^Departments of Biological Chemistry, ^2^Chemistry and ^3^Biophysics, University of Michigan Ann Arbor, MI 48109

^†^Equal contributors

**Running Title**: Patient mutations differentially impair ATR functions

*Address correspondence to: Ruma Banerjee, Tel: (734) 615-5238; E-mail: [rbanerje@umich.edu](mailto:rbanerje@umich.edu)

**Table of Contents**

1. Figure S1. Simulations of EPR spectra of cob(II)alamin bound to wild-type and E193K ATR.
2. Figure S2. Simulations of EPR spectra of cob(II)alamin bound to R190C and R190H ATR.


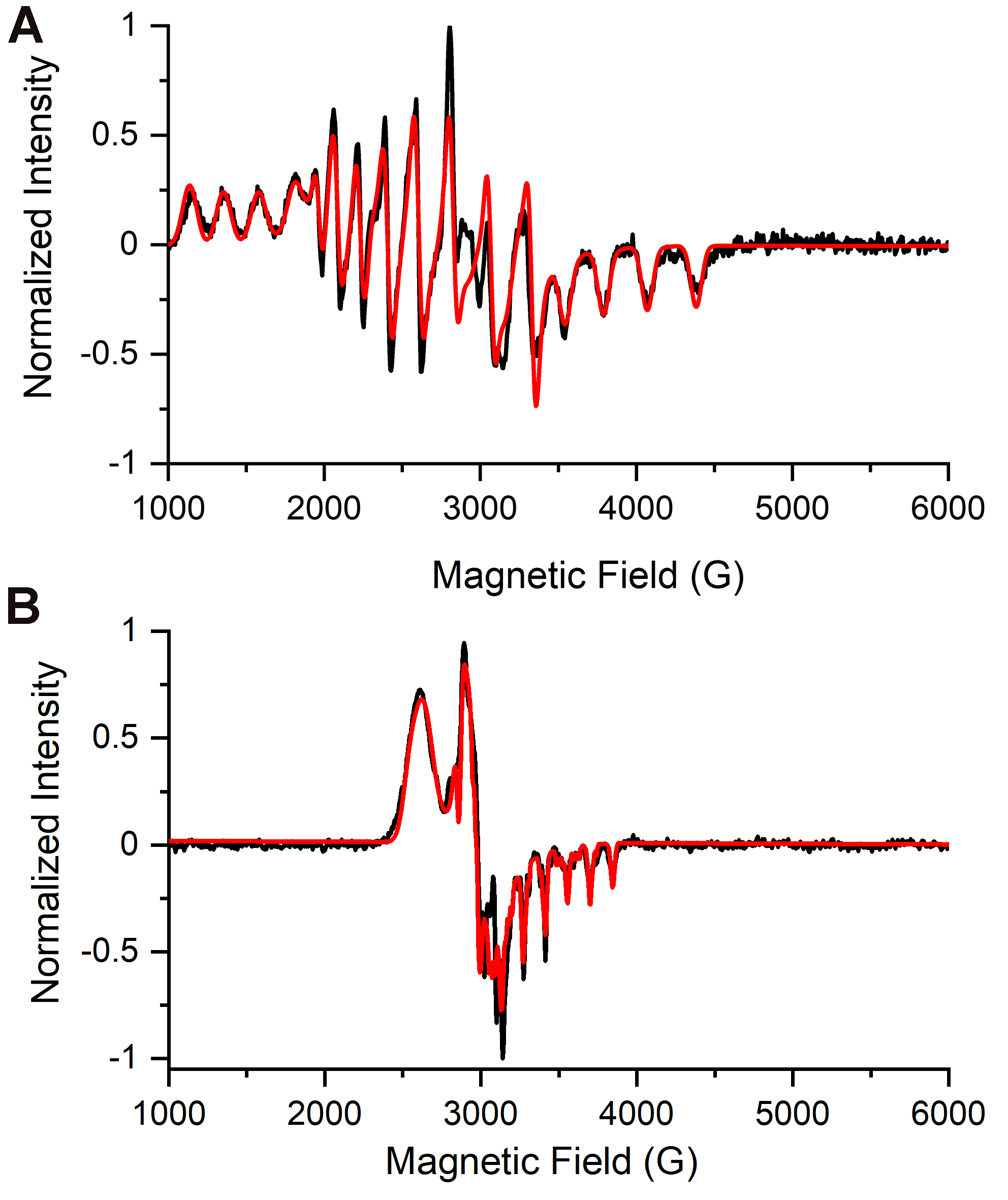


**Figure S1**. **Simulations of** **EPR spectra of cob(II)alamin bound to wild-type and E193K ATR. (A)** EPR spectrum of 4-coordinate base-off cob(II)alamin (300 μM) bound to wild-type ATR (200 μM trimer) in the presence of 5 mM ATP. The experimental and simulated spectra are shown in black and red respectively. The experimental spectrum was recorded at 100 K using the following parameters: 9.27 GHz microwave frequency, 20 mW power, 10 G modulation amplitude, 100 kHz modulation frequency, 5000 G sweep width centred at 3500 G, conversion time 164 msec, time constant 41 msec. Five scans were collected for each sample. The simulation was obtained using the following parameters: principal *g*-values, *g_x_*=3.32, *g*_y_=2.46, *g_z_*=1.75; principal hyperfine coupling constants, A_x_=1109, A_y_=666, A_z_=486 MHz. (**B)** EPR spectrum cob(II)alamin (300 μM) bound to ATR E193K (500 μM trimer) in the presence of 5 mM ATP. The experimental and simulated spectra are shown in black and red respectively. The experimental spectrum was recorded using the parameters described in A. The simulation was obtained using a mixture of 5-coordinate cob(II)alamin (76% base-off and 24% base-on) and the following parameters: 5-coordinate base-off cobalamin: principal *g*-values, *g_x_*=2.38, *g*_y_=2.30, *g_z_*=1.98; principal hyperfine coupling constants, A_x_=224, A_y_=186, A_z_=390 MHz; base-on cobalamin: principal *g*-values, *g_x_*=2.24, *g*_y_=2.21, *g_z_*=1.98; principal hyperfine coupling constants, A_x_=20, A_y_=20, A_z_=300, A_N_= 53 MHz)


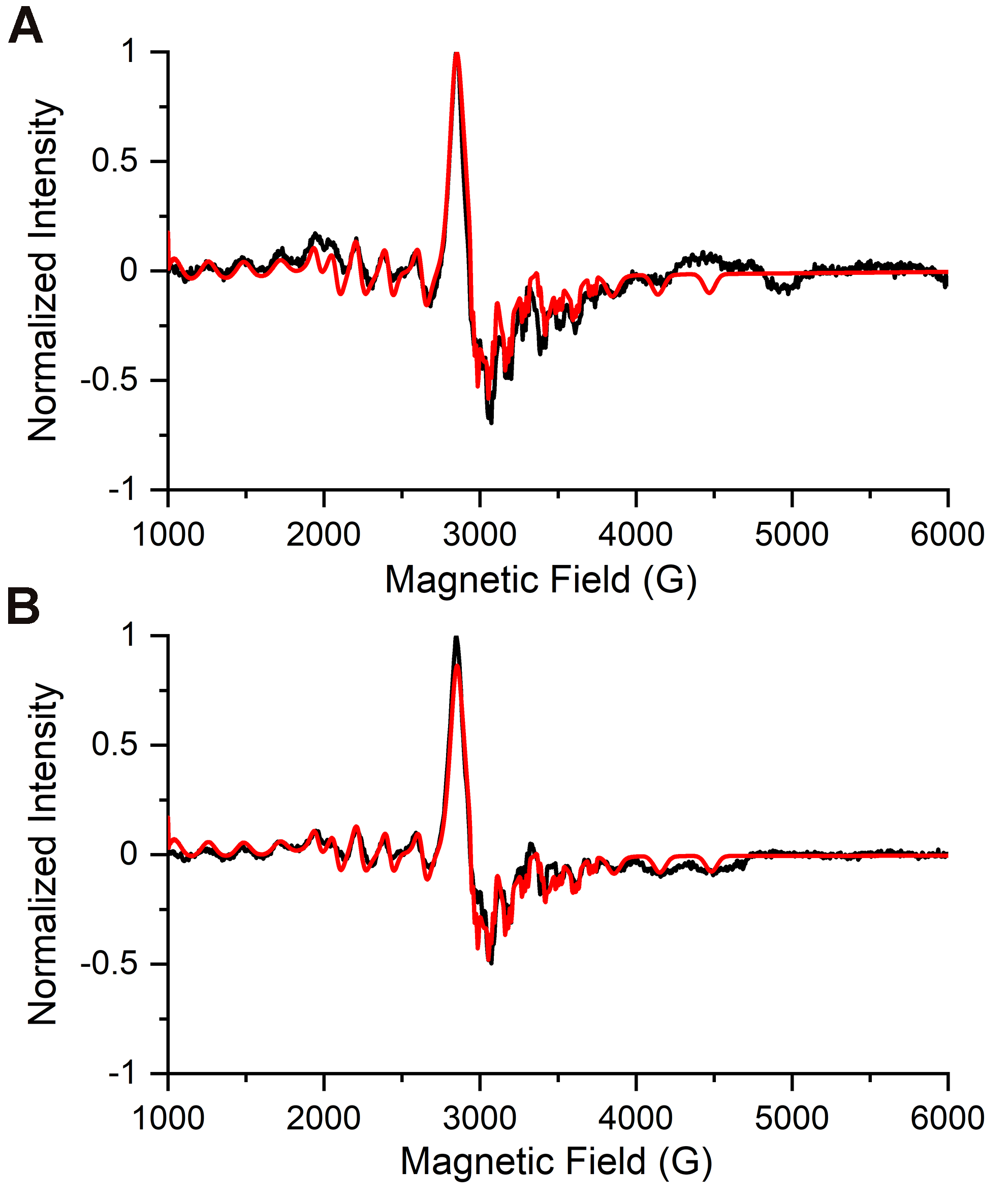


**Figure S2**. **Simulations of** **EPR spectra of cob(II)alamin bound to R190C and R190H ATR. (A,B)** EPR spectrum of cob(II)alamin (300 μM) bound to R190C (A) and R190H (B) ATR (500 μM each) in the presence of 5 mM ATP. The experimental and simulated spectra are shown in black and red respectively. The experimental spectrum was recorded using the parameters described in the Fig. S1 legend. The simulation was obtained with a mixture of 75% 4-coordinate and 25% 5-coordinate base-on cob(II)alamin using the following parameters: 4-coordinate cob(II)alalmin: *g_x_*=3.48, *g*_y_=2.43, *g_z_*=1.73; A_x_=1182, A_y_=694, A_z_=498 MHz. The parameters for 5-coordinate base-on cob(II)alamin were the same as described in Fig. S1 legend.
